# Non-Invasive Epigenetic Detection of Fetal Trisomy 21 in First Trimester Maternal Plasma

**DOI:** 10.1371/journal.pone.0027709

**Published:** 2011-11-23

**Authors:** Ji Hyae Lim, Shin Young Kim, So Yeon Park, Shin Yeong Lee, Mi Jin Kim, You Jung Han, Si Won Lee, Jin Hoon Chung, Moon Young Kim, Jae Hyug Yang, Hyun Mee Ryu

**Affiliations:** 1 Laboratory of Medical Genetics, Medical Research Institute, Cheil General Hospital and Women's Healthcare Center, Seoul, Korea; 2 Department of Obstetrics and Gynecology, Cheil General Hospital and Women's Healthcare Center, KwanDong University college of Medicine, Seoul, Korea; Aga Khan University, Pakistan

## Abstract

**Background:**

Down syndrome (DS) is the most common known aneuploidy, caused by an extra copy of all or part of chromosome 21. Fetal-specific epigenetic markers have been investigated for non-invasive prenatal detection of fetal DS. The phosphodiesterases gene, *PDE9A*, located on chromosome 21q22.3, is completely methylated in blood (*M-PDE9A*) and unmethylated in the placenta (*U-PDE9A*). Therefore, we estimated the accuracy of non-invasive fetal DS detection during the first trimester of pregnancy using this tissue-specific epigenetic characteristic of *PDE9A*.

**Methodology/Principal Findings:**

A nested, case-control study was conducted using maternal plasma samples collected from 108 pregnant women carrying 18 DS and 90 normal fetuses (each case was matched with 5 controls according to gestational weeks at blood sampling). All pregnancies were singletons at or before 12 weeks of gestation between October 2008 and May 2009. The maternal plasma levels of *M-PDE9A* and *U-PDE9A* were measured by quantitative methylation-specific polymerase chain reaction. *M-PDE9A* and *U-PDE9A* levels were obtained in all samples and did not differ between male and female fetuses. *M-PDE9A* levels did not differ between the DS cases and controls (1854.3 *vs* 2004.5 copies/mL; *P* = 0.928). *U-PDE9A* levels were significantly elevated in women with DS fetuses compared with controls (356.8 *vs* 194.7 copies/mL, *P*<0.001). The sensitivities of *U-PDE9A* level and the unmethylation index of *PDE9A* for non-invasive fetal DS detection were 77.8% and 83.3%, respectively, with a 5% false-positive rate. In the risk assessment for fetal DS, the adjusted odds ratios of *U-PDE9A* level and UI were 46.2 [95% confidence interval: 7.8–151.6] and 63.7 [95% confidence interval: 23.2–206.7], respectively.

**Conclusions:**

Our findings suggest that *U-PDE9A* level and the unmethylation index of *PDE9A* may be useful biomarkers for non-invasive fetal DS detection during the first trimester of pregnancy, regardless of fetal gender.

## Introduction

Prenatal testing is an integral component of obstetric practice. Detection of chromosomal aneuploidy has been considered the most common and important aspect of prenatal testing. Aneuploidy refers to changes in the numbers of chromosomes present inside a cell, and phenotypic effects are caused by an increased or decreased gene dosage as a result of gaining or losing a particular chromosome. The most common aneuploidy is trisomy 21, refered to as Down syndrome (DS), which occurs in 1 in 800 live births worldwide and is caused by an extra copy of all or part of chromosome 21 [1]. DS is associated with intellectual impairment, severe learning difficulties, and mortality caused by long-term health problems such as heart disease [1]. The incidence of fetal DS increases in a maternal age-dependent manner [1], [2].

Worldwide, millions of pregnant women undergo prenatal testing including screening tests and invasive diagnostic procedures for fetal DS detection every year. The current screening protocol for fetal DS involves a blood test for maternal protein biomarkers associated with DS (maternal serum alpha fetoprotein, unconjugated estriol, human chorionic gonadotrophin, and inhibin-A) as well as fetal ultrasound evaluations of nuchal translucency. However, the detection rate for each factor ranges from 25% to 68% with false-positive rate of 5% [3], [4]. Therefore, multiple screening tests are usually performed to increase the detection rate for fetal DS at 11–12 weeks and 16–18 weeks of gestation. However, such integrated tests have disadvantages such as increased cost due to the use of multiple biomarkers and psychological burden on the patient as they wait to obtain results in the second trimester (16–18 weeks of gestation). Moreover, 5% of normal pregnant women with false-positive results undergo unnecessary invasive diagnostic processes such as amniocentesis or chorionic villus sampling (CVS) for fetal DS detection. Such invasive diagnostic tests are expensive, require expert technicians, and carry a risk of miscarriage of approximately 1% [5], [6]. Therefore, non-invasive prenatal test that allow for the direct analysis of fetal genetic material in maternal blood have been investigated as alternative methods to reduce the need for invasive procedures and to improve the efficacy of current screening tests for fetal DS.

Cyclic nucleotide phosphodiesterases (PDEs) catalyze the hydrolysis of cyclic adenosine 30,50-monophosphate (cAMP) and/or cyclic guanosine 30,50-monophosphate (cGMP) [7]. The human *PDE9A* gene, located on chromosome 21q22.3, encodes a cGMP-specific PDE that is expressed in most tissues, but not in blood [8], [9]. Due to its location and contribution to the regulation of the steady-state cellular concentrations of cyclic nucleotides, *PDE9A* is a possible candidate gene for diseases such as bipolar affective disorder [9], and Guipponi *et al*. suggested that its overexpression might be involved in DS [9]. Recently, Chim *et al*. performed a systematic search for fetal-specific epigenetic markers on chromosome 21 [10], during which they found that the *PED9A* region is completely methylated in the maternal blood (*M-PED9A*) and unmethylated in fetal (placental) tissues (*U-PED9A*). Therefore, they reported that this marker was independent of fetal gender and genotype, and hence could be applied to all fetal-maternal pairs for non-invasive detection of fetal sequences on chromosome 21. However, the usefulness of *PED9A* in the non-invasive prenatal detection of fetal DS has not yet been clinically validated.

The aim of this study was to estimate the accuracy of non-invasive fetal DS detection using tissue specific-methylation in *PDE9A* and to evaluate the potential of *PDE9A* as a new biomarker for the first trimester detection of fetal DS.

## Materials and Methods

### Ethics statement

This study was conducted according to the principles expressed in the Declaration of Helsinki. Appropriate institutional review board approval was obtained from the Ethics Committee at Cheil General Hospital for this study (#CGH-IRB-2008-43). Written informed consent was obtained from each participant before blood draws for the collection of samples and subsequent analysis.

### Study participants and samples

We performed a nested case-control study of women who enrolled in the Cheil General Hospital Non-invasive Prenatal Diagnosis Study (CNPD). Participants in the CNPD were recruited from 2008 for non-invasive prenatal diagnosis of rare and incurable fetal diseases. Participants were women who received prenatal care at Cheil General Hospital. For the current study, 108 women were selected from a larger sample of 793 women enrolled in the CNPD according to criteria described below. All pregnancies were singletons at or before 12 weeks of gestation between October 2008 and May 2009.

The case group consisted of 18 women who were subsequently detected to be carrying a DS fetus by amniocentesis or CVS for fetal karyotyping. Each case was paired with five controls that were matched according to gestational week at blood sampling (Table S1). The control group (n = 90) included women who delivered healthy normal neonates at term (37 weeks of gestation or more) without medical or obstetric complications, such as hypertension, diabetes, renal insufficiency, congenital anomalies, or fetal demise. Before maternal blood sampling, ultrasonography was recommended to establish the viability of each singleton pregnancy and to confirm gestational age calculated from the time of last menstruation. Maternal, fetal, and infant records were collected prospectively and maintained in an electronic database. None of the participants had histories of preexisting hypertension, diabetes mellitus, liver disease, or chronic kidney disease.

### Isolation of Plasma and DNA

Ten milliliters of peripheral blood were collected from each participant into ethylenediaminetetraacetic acid (EDTA) tubes. Immediately after sampling, maternal peripheral blood samples were centrifuged at 1,600 g for 10 minutes at 4°C and the plasma portion was recentrifuged at 16,000 g for 10 minutes to minimize any additional release of maternal DNA. Circulating fetal DNA was extracted from 1.5 mL of maternal plasma using the QIAamp DSP Virus Kit (Qiagen, Hilden, Germany), according to the manufacturer's recommendations. Each DNA sample was eluted into 50 µL of sterile, DNase-free water. The samples were coded for subsequent blinded analysis.

### Quantitative methylation-specific polymerase chain reaction (qMSP)

**Figure 1:**
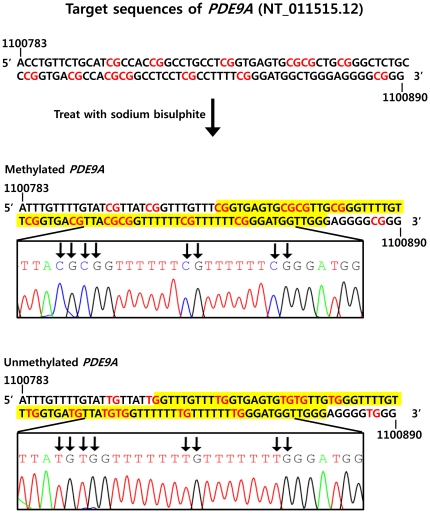
Bisulfite genomic sequencing of *PDE9A.* The red characters in the sequences indicate methylation CpG sites of the *PDE9A* gene. Methylated CpG sites of *PDE9A* were detected in maternal blood cells and placental tissues. Unmethylated CpG sites of *PDE9A* were detected only in placental tissues. Yellow boxes indicate the regions that were amplified by the methylation-specific polymerase chain reaction.

Differences in methylation of *PDE9A* in maternal blood cells and placenta were confirmed by bisulphite genomic sequencing (Fig. 1, Methods S1). Levels of *M-PDE9A* and *U-PDE9A* in maternal plasma were measured by qMSP assays as previously described [10], [11].

DNA extracted from plasma was bisulphite-converted using the EZ DNA Methylation Kit (Zymo Research, Orange, CA, USA) and then eluted into 30 µL. For the validation of bisulphite conversion, a synthetic DNA oligonucleotide for the *U-PDE9A* region and genomic DNA extracted from peripheral blood cells were used as positive and negative controls, respectively. The PCR reaction solution contained 12.5 µL iQ Supermix (Bio-Rad, Hercules, CA, USA), 200 nM hydrolysis probe (Applied Biosystems, Foster City, CA, USA), 400 nM primers (Applied Biosystems, Foster City, CA, USA), and 5 µL converted DNA per 25 µL total reaction volume. The primers and hydrolysis probes used for qMSP assay were as follows; for *M-PDE9A*, forward primer: 5′-CGG TGA GTG CGC GTC GC-3′, reverse primer: 5′-CCA ACC ATC CCG AAA AAG CG-3′, and probe: 5′-TTC GGT GAC GTT ACG CGG T-3′, for *U-PDE9A*, forward primer: 5′- GGT TTG TTT TGG TGA GTG TGT GTC GT-3′, reverse primer: 5′- CCC AAC CAT CCC AAA AAA GCA-3′, and probe: 5′-TTT GTT TGG TGA TGT TAT GTG GTT T-3′. The probes for *M-PDE9A* and *U-PDE9A* were dual-labeled with 6-carboxyfluorescein (FAM) and minor groove-binding non-fluorescent quencher (MGBNFQ). The thermal profile consisted of an initial denaturation step of 95°C for 10 minutes followed by 50 cycles of 95°C for 15 seconds, 60°C for 30 seconds, and 72°C for 30 seconds. Calibration curves were prepared for each assay by serial dilutions of single-stranded synthetic DNA oligonucleotides specific to the *M-PDE9A* and *U-PDE9A* amplicons. The sequences of the synthetic DNA were as follows; for *M-PDE9A*: 5′-CGG TGA GTG CGC GTT GCG GGT TTT GTT CGG TGA CGT TAC GCG GTT TTT TCG TTT TTT CGG GAT GGT TGG-3′, for *U-PDE9A*: 5′-GGT TTG TTT TGG TGA GTG TGT GTT GTG GGT TTT GTT TGG TGA TGT TAT GTG GTT TTT TTG TTT TTT TGG GAT GGT TGG G-3′.

Levels were calculated as copies/mL as previously described [12]. Strict precautions were taken to prevent contamination, and multiple negative-control water blanks were included in every analysis. To reduce inter-experimental variation, each extraction was repeated in triplicate. The final data reflect the means of the results (Table S2 and S3). All samples were analyzed blindly.

### Statistical Analysis

The clinical characteristics of the study population were compared between cases and controls using the Mann-Whitney U test and the χ^2^ test. The accuracy for detecting fetal DS was analyzed with factors such as *U-PDE9A* level and unmethylation index (UI). The UI is calculated as {*U-PDE9A* level/(*M-PDE9A* level+*U-PDE9A* level)}X100. The accuracy of fetal DS detection was determined with the fetal karyotyping results. Receiver operating characteristics (ROC) curve analysis was performed to assess the optimal cutoff value as described in our previous study [13]. The optimal cutoff was set at a 5% false-positive rate for comparisons with the accuracy of current screening test for fetal DS. The sensitivity, positive predictive value (PPV), negative predictive value (NPV), and odds ratio (OR) were calculated with 95% confidence intervals (CIs) using the EpiMax Table Calculator (http://www.healthstrategy.com/epiperl/epiperl.htm). Overall accuracy was estimated according to the area under the ROC curve (AUC). Multiple logistic regression analysis was used to estimate the values of *U-PDE9A* levels and UI as risk factors for fetal DS, controlling for potential confounding factors. Potential confounding factors included maternal age, body mass index, and nulliparity at the time of blood sampling. Adjusted ORs (adjORs) and their 95% CIs were calculated. In all tests, thresholds of *P*<0.05 were set for statistical significance. Statistical analyses were performed using the Statistical Package for Social Sciences 12.0 (SPSS Inc., Chicago, IL, USA).

## Results

The clinical characteristics are given in Table 1. At blood sampling, maternal age, gravidity, and body mass index did not significantly differ between the cases and controls (*P*>0.05 for all). The median gestational age was 7.5 weeks in both case and control groups. Forty-four percent of participants in the case group and 41% of participants in the control group were nulliparous; this difference between cases and controls was not significant (*P* = 0.799). Fetal gender-ratio and tobacco use also were not different between the two groups (*P*>0.05 for both).

**Table 1:** Clinical characteristics in cases and controls.

Characteristics	Case (N = 18)	Control (N = 90)	*P* value
At blood sampling			
Maternal age (years)	36.1 (31.3–38.0)	31.1 (29.1–34.8)	0.059^ a^
Gestational age (weeks)	7.5 (6.0–9.0)	7.5 (6.0–9.0)	0.999^ a^
Body mass index (kg/m^2^)	22.2 (20.6–24.0)	21.4 (19.3–23.3)	0.180^ a^
Nulliparity	8 (44.4)	37 (41.1)	0.799^ b^
Gravidity	2.0 (1.0–3.3)	2.0 (2.0–3.0)	0.331^ a^
Gender-ratio of fetus (male:female)	7∶11	44∶46	0.606^ b^
Tobacco use	1 (5.6)	6 (6.7)	0.999^ b^

Values are median (interquartile range) or number (%).

a: Mann-Whitney U test, b: χ^2^ test

**Figure 2:**
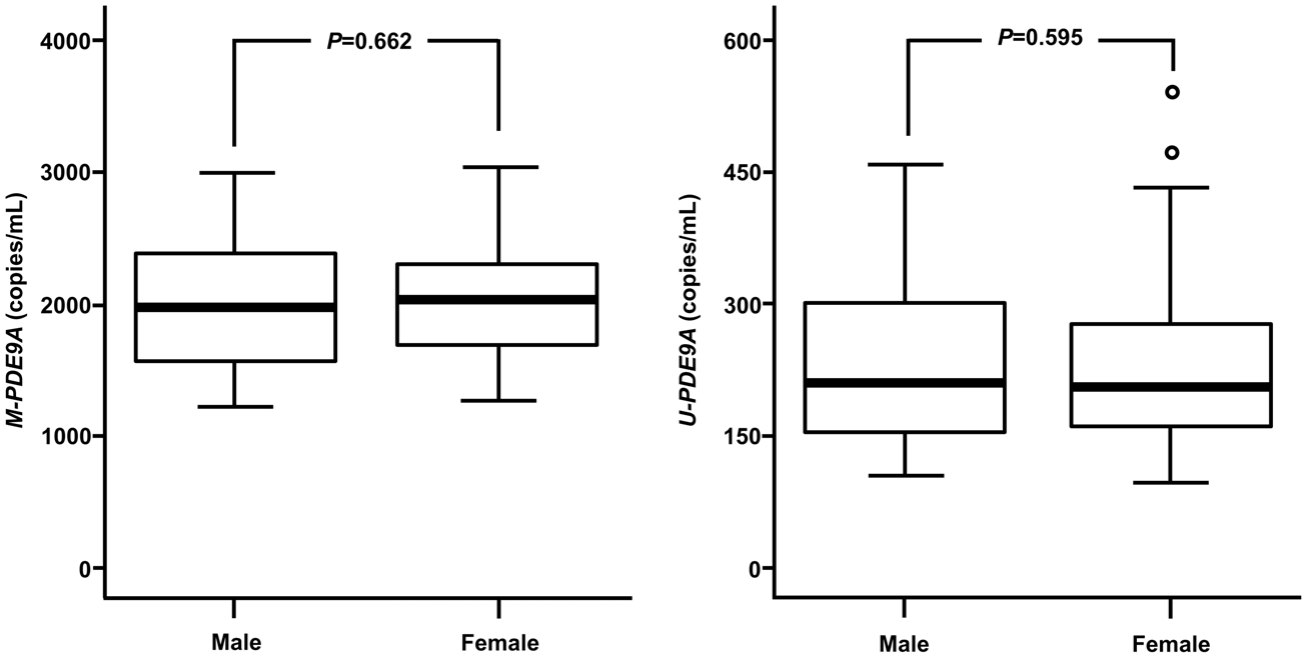
Levels of *M-PDE9A* and *U-PDE9A* in maternal plasma according to fetal gender. The upper and lower limits of the boxes and the lines across the boxes indicate the 75^th^/25^th^ percentiles and the medians, respectively. The upper and lower error bars indicate the 90^th^ and 10^th^ percentiles, respectively. The circles indicate outliers. Levels were analyzed by the Mann-Whitney U-test.

**Figure 3:**
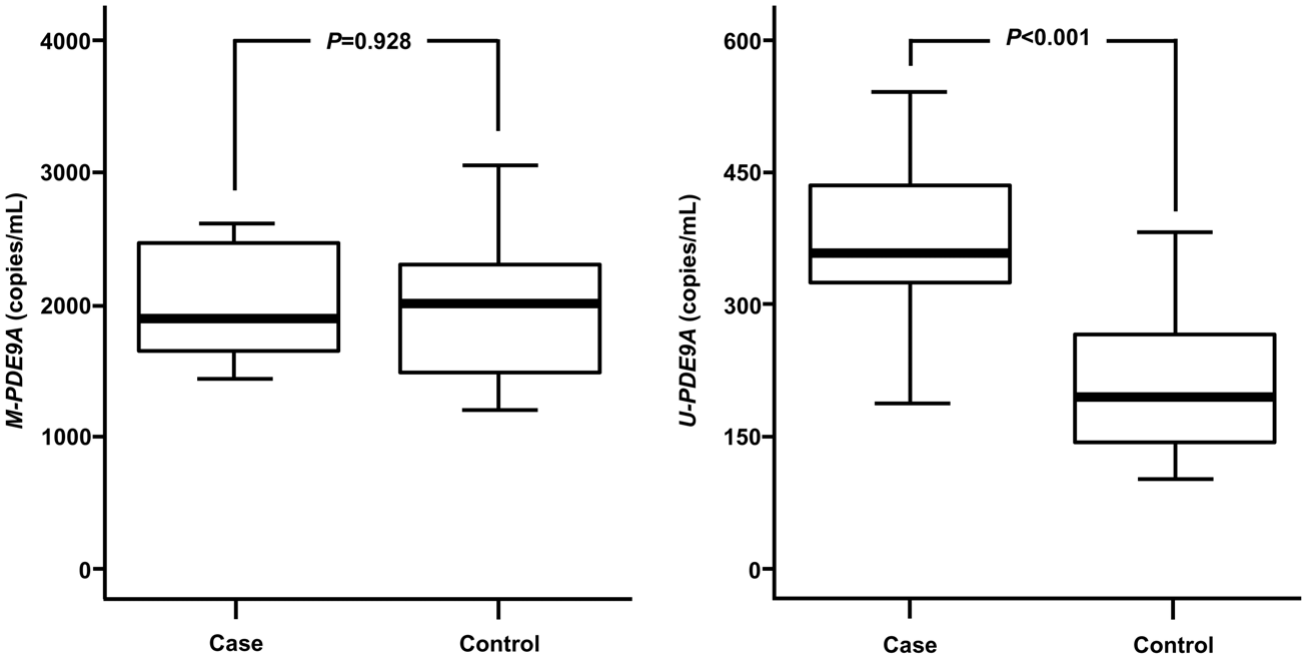
Levels of *M-PDE9A* and *U-PDE9A* in maternal plasma according to fetal karyotype. The upper and lower limits of the boxes and the lines across the boxes indicate the 75^th^/25^th^ percentiles and the medians, respectively. The upper and lower error bars indicate the 90^th^ and 10^th^ percentiles, respectively. Levels were analyzed by the Mann-Whitney U-test.

In the analysis of *M-PDE9A* and *U-PDE9A* levels according to fetal gender, median levels of *M-PDE9A* were 1956.0 (interquartile range: 1592.5–2411.2) and 2023.1 (interquartile range: 1650.0–2310.3) copies/mL in male and female fetus-bearing participants, respectively. Levels of *U-PDE9A* were 213.6 (interquartile range: 155.1–308.4) and 209.6 (interquartile range: 156.3–283.5) copies/mL in male and female fetus-bearing participants, respectively. Levels of *M-PDE9A* and *U-PDE9A* were not significantly different between fetal genders (*P*>0.05 for all, Fig. 2). In the analysis of *M-PDE9A* and *U-PDE9A* levels according to fetal karyotype, levels of *M-PDE9A* were not different between the DS cases and controls [1854.3 (interquartile range: 1653.3–2446.3) *vs* 2004.5 (interquartile range: 1605.2–2316.9)copies/mL, *P* = 0.928, Fig. 3]. However, levels of *U-PDE9A* were significantly higher in the cases compared to controls [356.8 (interquartile range: 314.8–437.4) *vs* 194.7 (interquartile range: 143.0–263.6) copies/mL, *P*<0.001, Fig. 3].

**Figure 4:**
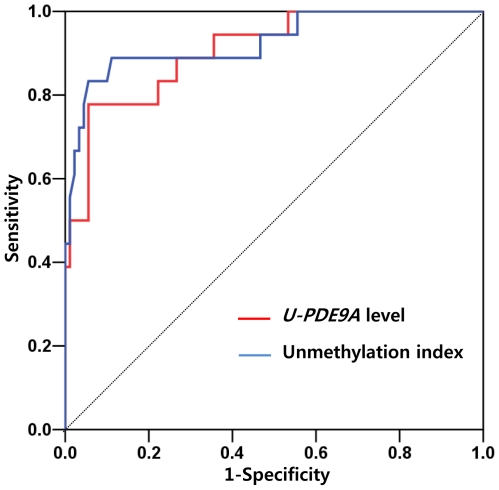
The ROC curves of *U-PDE9A* level and unmethylation index. The ROC curves of unmethylation index and *U-PDE9A* level are represented by blue and red lines, respectively.

The accuracies of *U-PDE9A* level and UI for the detection of fetal DS are shown in Table 2. The cutoff value for each factor was set at the 5% false-positive rate by ROC analysis. Sensitivity, PPV, and NPV were compared at an equivalent false-positive rate. The ROC curves of *U-PDE9A* level and UI are presented in Fig. 4. The *U-PDE9A* cutoff level of 320 copies/mL had a sensitivity of 77.8%, PPV of 73.7%, and NPV of 95.5% in differentiating women carrying a DS fetus from women carrying a normal fetus, and the AUC was 0.907 (95% CI: 0.833–0.981) with a standard error (SE) of 0.038 (*P*<0.001). The UI cutoff value of 14 had a sensitivity of 83.3%, PPV of 75.0%, NPV of 96.6%, and the AUC was 0.927 (95% CI: 0.851–1.003) with an SE of 0.039 (*P*<0.001). These findings were confirmed using sample karyotypes (Table S1).

**Table 2:** The utility of measurements for detection of fetal Down syndrome.

	Cutoff	Sensitivity	PPV	NPV
*U-PDE9A* level (copies/mL)	320	0.778(0.557–0.891)	0.737(0.537–0.863)	0.955(0.912–0.982)
Unmethylation index	14	0.833(0.635–0.940)	0.750(0.563–0.855)	0.966(0.923–0.990)

The cutoff was set at 5% false positive rate by ROC curve analysis. To compare detection accuracies of factors, sensitivity, positive predictive value (PPV), and negative predictive value (NPV) were calculated at equivalent false positive rate. The number in parentheses indicates the 95% confidence interval.

We analyzed the accuracy of *U-PDE9A* levels and UI for detecting fetal DS based on gestational weeks at blood sampling (Table 3). The sensitivity of *U-PDE9A* level was higher at 9–12 weeks of gestation than at 5–8 weeks of gestation, whereas the sensitivity of UI was higher at 5–8 weeks of gestation than at 9–12 weeks of gestation. The specificities of both tests were higher at 9–12 weeks of gestation than at 5–8 weeks of gestation. All cases showing false-positive and false-negative results were observed at or before a gestational age of 10 weeks (Table 4).

**Table 3:** Detection accuracy according to gestational weeks.

	Gestational weeks	Case(correct N/total N)	Control(correct N/total N)	Sensitivity	Specificity	PPV	NPV
*U-PDE9A*Level	5–8	10/13	61/65	0.769(0.509–0.925)	0.938(0.886–0.970)	0.714(0.472–0.859)	0.953(0.900–0.985)
	9–12	4/5	24/25	0.800(0.347–0.982)	0.960(0.869–0.996)	0.800(0.347–0.982)	0.960(0.869–0.996)
Unmethylation index	5–8	11/13	61/65	0.846(0.588–0.970)	0.938(0.887–0.963)	0.733(0.510–0.841)	0.968(0.915–0.994)
	9–12	4/5	24/25	0.800(0.347–0.982)	0.960(0.869–0.996)	0.800(0.347–0.982)	0.960(0.869–0.996)

PPV, positive predictive value; NPV, negative predictive value.

The number in parentheses indicates the 95% confidence interval.

**Table 4:** Cases showing false-positive and false-negative results.

Factors	Condition	Value	Gestational weeks	Karyotype
*U-PDE9A* level(cutoff: 320 copies/mL)	False-negative(N = 4)	228.4	6	47,XX,+21
		280.6	6	47,XX,+21
		186.6	7	47,XY,+21
		255.9	9	47,XX,+21
	False-positive(N = 5)	361.6	6	46,XX
		381.1	7	46,XY
		345.9	7	46,XX
		351.8	8	46,XX
		366.5	10	46,XY
Unmethylation index (cutoff: 14)	False-negative(N = 3)	9.5	6	47,XX,+21
		8.7	7	47,XY,+21
		12.9	9	47,XX,+21
	False-positive(N = 5)	16.5	6	46,XY
		15.8	7	46,XX
		14.9	8	46,XY
		14.4	8	46,XX
		15.3	10	46,XY

We analyzed the associations between each factor and the risk of DS. As listed in Table 5, the risk of DS was significantly increased in women with values greater than the cutoff values compared with women whose values were less than the cutoff values for *U-PDE9A* level or UI (OR [95% CI]: 59.5 [12.1–340.7] for *U-PDE9A* level, 85.0 [15.5–564.2] for UI). After adjusting for potential confounding factors such as maternal age, body mass index, and nulliparity at blood sampling, the adjORs of *U-PDE9A* level and UI were 46.2 (95% CI: 7.8–151.6) and 63.7 (95% CI: 23.2–206.7), respectively. Women with *U-PDE9A* levels and UI greater than the cutoff values were at dramatically higher risk of fetal DS in both unadjusted and adjusted analyses, compared with women with values less than the cutoff values.

**Table 5:** Odds ratios for fetal Down syndrome.

	Case(N = 18)	Control(N = 90)	Unadjusted OR (95%CI)	Adjusted OR(95% CI)*
*U-PDE9A* level (copies/mL)				
Lower than 320	4	85	1.0	1.0
320 or higher	14	5	59.5 (12.1–340.7)	46.2 (7.8–151.6)
Unmethylation index				
Lower than 14	3	85	1.0	1.0
14 or higher	15	5	85.0 (15.5–564.2)	63.7 (23.2–206.7)

OR, odds ratio; CI, confidence interval.

*Adjusted for maternal age, body mass index, and nullipara at blood sampling

## Discussion

Our findings demonstrate that *U-PDE9A* and UI are effective biomarkers for the non-invasive detection of fetal DS during the first trimester of pregnancy. We determined that *U-PDE9A* levels and UI were significantly elevated in pregnant women carrying DS fetuses compared to women carrying normal fetuses in the first trimester, regardless of fetal gender. Moreover, pregnant women with high values for *U-PDE9A* and UI were at an increased risk of carrying a DS fetus. Therefore, we suggest that *U-PDE9A* and UI may be useful biomarkers for non-invasive fetal DS detection using circulating fetal DNA from maternal plasma, regardless of fetal gender.

Current screening tests for fetal DS detection increases detection rate by integration of multiple methods, such as measurement of nuchal translucency and blood tests for numerous maternal serum markers, including alpha fetoprotein, unconjugated estriol, human chorionic gonadotrophin, and inhibin-A, but at a higher screen-positive rate (approximately 16–22%) [3], [4]. Moreover, diagnostic test currently requires obtaining samples of fetal cells directly from the uterus for genetic analysis, either through CVS between 11 and 14 weeks gestation or amniocentesis after 15 weeks. However, these invasive procedures present a small but significant risk of miscarriage in normal pregnancies [5], [6]. Therefore, the development of non-invasive prenatal tests for DS detection using circulating fetal DNA in maternal plasma has been considered as a potential alternative to improve current screening tests and to reduce the need for invasive procedures, such as amniocentesis or CVS.

Epigenetics is the study of molecular phenomena that affect gene expression, but which do not involve alterations in DNA sequences. The best-studied epigenetic phenomenon is the process of DNA methylation, which suppresses gene expression and exhibits tissue-specific patterns [14]. In a prior study, Poon *et al.* demonstrated that it was possible to develop fetal DNA markers based on differential DNA methylation patterns between maternal and fetal tissues [15]. This strategy has led to the recognition that the *SERPINB5* gene on chromosome 18, which codes for maspin, is hypomethylated in the placenta, but hypermethylated in maternal blood cells [15]. Therefore, it has been suggested that the measurement of the ratio of single nucleotide polymorphisms (SNP) in the hypomethylated version of the *SERPINB5* gene might allow the detection of fetal trisomy 18 [16].

Similar studies have been performed for non-invasive prenatal detection of fetal DS using circulating fetal DNA. Recently, Papageorgiou *et al.* reported that the application of fetal-specific methylation ratio led to correct diagnosis of fetal DS [17] and Tong *et al.* reported that epigenetic-genetic chromosome dosage using hypermethylated *HLCS* and a paternally inherited fetal SNP allele could be applied for the prenatal diagnosis of trisomy 21 for both male and female fetuses [18]. However, these studies were performed with maternal blood samples that were taken during the late-first or second trimesters (11.1–14.4 weeks of gestation in Papageorgiou *et al.* [17]; 12–21 weeks of gestation in Tong *et al.* [18]) and the samples ware small (14 cases and 26 controls in a blinded analysis by Papageorgiou *et al.* [17]; 14 cases and 33 controls in Tong *et al.* [18]). Moreover, these studies did not control for gestational weeks at blood sampling, which can affect the concentrations of circulating fetal DNA [17], [18]. Despite these limitations, previous studies suggest that diagnostic strategies using fetal-specific epigenetic markers may be advantageous compared to current biochemical screening methods for fetal DS. Therefore, searches for fetal-specific epigenetic markers on chromosome 21 are important for non-invasive prenatal detection of fetal DS. Recently, Chim *et al.* performed a systematic search for such markers on chromosome 21 [10] and reported that 22 of the 114 studied genomic regions were differentially methylated between maternal and fetal (placental) tissues. On the basis of this tissue-specific methylation, they proposed the use of fetal-DNA markers, such as *U-PDE9A*, which are completely methylated in maternal blood cells but unmethylated in the placenta for non-invasive prenatal detection of fetal sequences on chromosome 21. However, the usefulness of such markers in non-invasive prenatal test for fetal DS has not yet been confirmed.

In the present study, we investigated the utility and accuracy of *U-PDE9A* levels and UI for use in non-invasive prenatal detection of DS using circulating fetal DNA from the first trimester. We conducted a nested case-control study controlling for gestational weeks at blood sampling; each case was matched carefully with 5 controls according to gestational weeks at blood sampling (Table S1). Furthermore, all samples were obtained during the first trimester (12 weeks of gestation or less) and the sample number was larger than those of prior studies [17], [18]. In this study, *U-PDE9A* levels and UI were significantly elevated during the first trimester in pregnant women carrying DS fetuses, regardless of fetal gender. The fetal DS detection rates of the two factors were higher than those of serum screening markers and nuchal translucency measurements, which are generally performed in the first trimester [3], [4]. Moreover, the detection rates of *U-PDE9A* and UI for fetal DS were far superior to those of Y chromosome-specific markers, such as *DYS14* or *SRY*, in maternal serum [19]. Furthermore, Y chromosome-specific markers are limited to use for male fetuses. Therefore, we suggest that *U-PDE9A* levels and UI are effective markers for use in non-invasive prenatal detection of DS during the first trimester regardless of fetal gender, and in fact, these markers may detect fetal DS more effectively than current screening tests for fetal DS. However, the sensitivities of *U-PDE9A* level and the UI were lower or similar (77.8% for *U-PDE9A* levels and 83.3% for UI) than those of second trimester quadruple screening tests (81%). Moreover, false-positive and false-negative results were observed at or before 10 weeks of gestational age. Accordingly, non-invasive fetal DS detection using these markers may have limited clinical applications prior to 10 weeks of gestation. Therefore, improvements in detection rate by applications of other detection methods such as digital PCR or direct comparisons with fetal-specific DNA markers on reference chromosomes may facilitate the clinical use of these markers.

In non-invasive prenatal testing, the analysis of fetal genetic loci in maternal plasma is still problematic because circulating fetal DNA is present in small amounts. To solve this problem, many researchers have suggested methods such as size fractionation [20], [21]. Prior studies have demonstrated that more than 99% of circulating fetal DNA is less than 313 bp in length, whereas circulating maternal DNA is significantly longer [20], [21]. This discovery enables the possibility of circulating fetal DNA enrichment by size fractionation using a number of methods. Most simply, gel electrophoresis of DNA extracted from maternal plasma has been used to select for low molecular weight DNA. Other methods include the use of kits and columns that rely either on the inability of large molecular weight DNA to pass through, or by the retention of low molecular weight DNA [22], [23]. In recent studies, Clausen *et al.* reported that using the DSP Virus Kit resulted in a higher yield of circulating fetal DNA compared with total circulating DNA from maternal plasma [22]. Legler *et al.* suggested that the DSP Virus Kit is an optimal method for extracting high yields of circulating fetal DNA from total plasma [23]. In this study, we used the DSP Virus Kit to extract circulating fetal DNA from maternal plasma. Therefore, we were able to detect fetal DNA identifiers, such as *U-PDE9A*, at as early as five weeks of gestation.

To our knowledge, this is the first study to estimate the accuracy of non-invasive fetal DS detection using *U-PDE9A* analysis in circulating fetal DNA from first trimester maternal plasma. Our data indicate that *U-PDE9A* and UI may be useful markers for non-invasive prenatal detection for fetal DS in all fetal-maternal pairs, regardless of fetal gender. Additionally, high *U-PDE9A* levels and UI may be suitable biomarkers for identifying pregnant women carrying fetuses with DS. Accordingly, these markers may be useful in the development of effective and reliable tests for non-invasive detection of fetal DS. However, the use of this method may create ethical and social issues because the results may impact decisions to terminate or continue the pregnancy. Therefore, the use of this method should be carefully considered in clinical situation and should not be used in the present clinical settings unless more research is done. Furthermore, this method needs to be further refined to achieve higher sensitivity and specificity. Additionally, this study is limited by its small sample size and inclusion of only Korean patients. Therefore, a larger-scale study within different ethnic populations will need to be performed to assist the introduction of the diagnostic strategy used in this study.

## Supporting Information

Methods S1 **There are included sample processing and DNA extraction for bisulfite genomic sequencing of** ***PDE9A*** **, bisulfite genomic sequencing of** ***PDE9A*** **, and limit of detection for quantitative methylation-specific polymerase chain reaction.**
(DOC)

Table S1 **DNA samples from participants in this study.**
(DOC)

Table S2 **Unmethylated** ***PDE9A*** **levels obtained from 108 samples.**
(DOC)

Table S3 **Methylated** ***PDE9A*** **levels obtained from 108 samples.**
(DOC)
