# Supplementary material for: Non-Invasive Epigenetic Detection of Fetal Trisomy 21 in First Trimester Maternal Plasma
Source: PLoS One. 2011 Nov 23;6(11):e27709. doi: 10.1371/journal.pone.0027709 (PMC3223183; doi:10.1371/journal.pone.0027709)
Supplement: Methods S1 — There are included sample processing and DNA extraction for bisulfite genomic sequencing of PDE9A , bisulfite genomic sequencing of PDE9A , and limit of detection for quantitative methylation-specific polymerase chain reaction. (DOC) [file pone.0027709.s001.doc]

**Supplementary Methods**

*Sample processing and DNA extraction for bisulfite genomic sequencing of PDE9A*

Maternal peripheral blood samples and placental tissues for bisulfite genomic sequencing of *PDE9A* were collected from healthy women (n=5) who delivered normal neonates at term (37 weeks of gestation or more) without medical or obstetric complications. Maternal peripheral blood samples (10 mL) and placental tissues (5 g) were collected at 24 hr before the delivery and immediately after the delivery, respectively. Maternal peripheral blood samples were collected into tubes containing EDTA just before the obstetric procedures. Maternal peripheral blood samples were centrifuged at 1,600g for 10 min at 4°C. The peripheral blood cell portion was recentrifuged at 2,500g and any residual plasma was removed. DNA was extracted from peripheral blood cells with the Nucleon blood DNA extraction kit (GE Healthcare), and from placental tissues with the QIAamp tissue kit (Qiagen) according to the manufacturer’s recommendations. Informed consent was obtained from women. The study was approved by the institutional review board in the Ethics Committee at Cheil General Hospital.

*Bisulfite genomic sequencing of PDE9A*

Each DNA sample extracted from maternal blood cells and placental tissues was subjected to bisulfite conversion using the CpGenome universal DNA modification kit (Chemicon) according to the manufacturer’s instructions. The bisulfite-treated DNA was then amplified by PCR with primers that did discriminate between the methylated or unmethylated CpG sites of *PDE9A* gene. The primers used for sequencing were as follows; for *M-PDE9A*, forward primer: 5’-CGG TGA GTG CGC GTC GC-3’, reverse primer: 5’-CCA ACC ATC CCG AAA AAG CG-3’; for *U-PDE9A*, forward primer: 5’- GGT TTG TTT TGG TGA GTG TGT GTC GT-3’, reverse primer: 5’- CCC AAC CAT CCC AAA AAA GCA-3’. PCR reaction solutions contained 10 ng genomic DNA, 10 pM primers, 0.25 mM dNTPs, 1.5 mM MgCl2, 1 X buffer, and 0.25 U Taq polymerase per 50 µL of total reaction volume. PCR conditions included predenaturation at 94°C for 5 min, 35 cycles of 94°C for 45 sec, 60°C for 45 sec, 72°C for 45 sec, and final extension at 60°C for 30 min.

After PCR amplification, PCR products were purified using a PCR purification kit (BIONEER, Korea) and sequenced using a PRISM BigDye Terminator Cycle Sequencing Kit (Applied Biosystems Inc., USA). Sequencing products were analyzed using a PRISM 3100 Genetic Analyzer (Applied Biosystems Inc., USA) and electropherogram traces were interpreted with Genescan software version 3.7 (Applied Biosystems Inc., USA). Corresponding genotypes were assigned using Genotyper software version 3.7 (Applied Biosystems Inc., USA).

*Limit of detection for quantitative methylation-specific polymerase chain reaction*

The mean fetal DNA concentration in maternal plasma was previously estimated to be 25.4 copies/mL (range 3.3-69.4) in early pregnancy [1]. In this study, 1.5 mL of maternal plasma is extracted, eluted in 30 µL, then 5 µL is used for each PCR. Thus, each PCR has fetal DNA of more than 6 copies. Therefore, a PCR is sensitive enough; at least 3 copies of the target are required at a qMSP.

Reference

1. Lo YM, Tein MS, Lau TK, Haines CJ, Leung TN, et al. (1998) Quantitative analysis of fetal DNA in maternal plasma and serum: implications for non-invasive prenatal diagnosis. Am J Hum Genet 62:768-775.
