# Supplementary material for: Non-Invasive Epigenetic Detection of Fetal Trisomy 21 in First Trimester Maternal Plasma
Source: PLoS One. 2011 Nov 23;6(11):e27709. doi: 10.1371/journal.pone.0027709 (PMC3223183; doi:10.1371/journal.pone.0027709)
Supplement: Table S1 — DNA samples from participants in this study. (DOC) [file pone.0027709.s002.doc]

**Supplementary Table 1.** DNA samples participated in the study

| Sample | Status | Gestational weeks  at blood sampling | Karyotype |
| --- | --- | --- | --- |
| P12 | Trisomy 21 | 5 | 47,XY,+21 |
| P19 | Normal | 5 | 46,XX |
| P20 | Normal | 5 | 46,XX |
| P21 | Normal | 5 | 46,XX |
| P65 | Normal | 5 | 46,XY |
| P66 | Normal | 5 | 46,XY |
| P1 | Trisomy 21 | 6 | 47,XX,+21 |
| P2 | Trisomy 21 | 6 | 47,XX,+21 |
| P3 | Trisomy 21 | 6 | 47,XX,+21 |
| P13 | Trisomy 21 | 6 | 47,XY,+21 |
| P22 | Normal | 6 | 46,XX |
| P23 | Normal | 6 | 46,XX |
| P24 | Normal | 6 | 46,XX |
| P25 | Normal | 6 | 46,XX |
| P26 | Normal | 6 | 46,XX |
| P27 | Normal | 6 | 46,XX |
| P28 | Normal | 6 | 46,XX |
| P29 | Normal | 6 | 46,XX |
| P30 | Normal | 6 | 46,XX |
| P31 | Normal | 6 | 46,XX |
| P67 | Normal | 6 | 46,XY |
| P68 | Normal | 6 | 46,XY |
| P69 | Normal | 6 | 46,XY |
| P70 | Normal | 6 | 46,XY |
| P71 | Normal | 6 | 46,XY |
| P72 | Normal | 6 | 46,XY |
| P73 | Normal | 6 | 46,XY |
| P74 | Normal | 6 | 46,XY |
| P75 | Normal | 6 | 46,XY |
| P76 | Normal | 6 | 46,XY |
| P4 | Trisomy 21 | 7 | 47,XX,+21 |
| P5 | Trisomy 21 | 7 | 47,XX,+21 |
| P14 | Trisomy 21 | 7 | 47,XY,inv(9)(p11q13),+21 |
| P15 | Trisomy 21 | 7 | 47,XY,+21 |
| P32 | Normal | 7 | 46,XX |
| P33 | Normal | 7 | 46,XX |
| P34 | Normal | 7 | 46,XX |
| P35 | Normal | 7 | 46,XX |
| P36 | Normal | 7 | 46,XX |
| P37 | Normal | 7 | 46,XX |
| P38 | Normal | 7 | 46,XX |
| P39 | Normal | 7 | 46,XX |
| P40 | Normal | 7 | 46,XX |
| P41 | Normal | 7 | 46,XX |
| P77 | Normal | 7 | 46,XY |
| P78 | Normal | 7 | 46,XY |
| P79 | Normal | 7 | 46,XY |
| P80 | Normal | 7 | 46,XY |
| P81 | Normal | 7 | 46,XY |
| P82 | Normal | 7 | 46,XY |
| P83 | Normal | 7 | 46,XY |
| P84 | Normal | 7 | 46,XY |
| P85 | Normal | 7 | 46,XY |
| P86 | Normal | 7 | 46,XY |
| P6 | Trisomy 21 | 8 | 47,XX,+21 |
| P7 | Trisomy 21 | 8 | 47,XX,+21 |
| P8 | Trisomy 21 | 8 | 47,XX,+21 |
| P16 | Trisomy 21 | 8 | 48,XXY,+21 |
| P42 | Normal | 8 | 46,XX |
| P43 | Normal | 8 | 46,XX |
| P44 | Normal | 8 | 46,XX |
| P45 | Normal | 8 | 46,XX |
| P46 | Normal | 8 | 46,XX |
| P47 | Normal | 8 | 46,XX |
| P48 | Normal | 8 | 46,XX |
| P49 | Normal | 8 | 46,XX |
| P50 | Normal | 8 | 46,XX |
| P51 | Normal | 8 | 46,XX |
| P52 | Normal | 8 | 46,XX |
| P53 | Normal | 8 | 46,XX |
| P54 | Normal | 8 | 46,XX |
| P55 | Normal | 8 | 46,XX |
| P87 | Normal | 8 | 46,XY |
| P88 | Normal | 8 | 46,XY |
| P89 | Normal | 8 | 46,XY |
| P90 | Normal | 8 | 46,XY |
| P91 | Normal | 8 | 46,XY |
| P92 | Normal | 8 | 46,XY |
| P9 | Trisomy 21 | 9 | 47,XX,+21 |
| P56 | Normal | 9 | 46,XX |
| P93 | Normal | 9 | 46,XY |
| P94 | Normal | 9 | 46,XY |
| P95 | Normal | 9 | 46,XY |
| P96 | Normal | 9 | 46,XY |
| P10 | Trisomy 21 | 10 | 47,XX,+21 |
| P57 | Normal | 10 | 46,XX |
| P58 | Normal | 10 | 46,XX |
| P59 | Normal | 10 | 46,XX |
| P97 | Normal | 10 | 46,XY |
| P98 | Normal | 10 | 46,XY |
| P17 | Trisomy 21 | 11 | 47,XY,+21 |
| P60 | Normal | 11 | 46,XX |
| P61 | Normal | 11 | 46,XX |
| P99 | Normal | 11 | 46,XY |
| P100 | Normal | 11 | 46,XY |
| P101 | Normal | 11 | 46,XY |
| P11 | Trisomy 21 | 12 | 47,XX,+21 |
| P18 | Trisomy 21 | 12 | 47,XY,+21 |
| P62 | Normal | 12 | 46,XX |
| P63 | Normal | 12 | 46,XX |
| P64 | Normal | 12 | 46,XX |
| P102 | Normal | 12 | 46,XY |
| P103 | Normal | 12 | 46,XY |
| P104 | Normal | 12 | 46,XY |
| P105 | Normal | 12 | 46,XY |
| P106 | Normal | 12 | 46,XY |
| P107 | Normal | 12 | 46,XY |
| P108 | Normal | 12 | 46,XY |
