# Supplementary material for: Non-Invasive Epigenetic Detection of Fetal Trisomy 21 in First Trimester Maternal Plasma
Source: PLoS One. 2011 Nov 23;6(11):e27709. doi: 10.1371/journal.pone.0027709 (PMC3223183; doi:10.1371/journal.pone.0027709)
Supplement: Table S2 — Unmethylated PDE9A levels obtained from 108 samples. (DOC) [file pone.0027709.s003.doc]

**Supplementary Table 2.** Fetal-specific unmethylated *PDE9A* levels obtained from the 108 samples

| Sample | Status | Levels | Inter SD | Inter CV | Intra SD | | Intra CV | Gender |
| --- | --- | --- | --- | --- | --- | --- | --- | --- |
| P1 | Trisomy 21 | 228.4 | 3.8 | 0.017 | | 5.3 | 0.023 | F |
| P2 | Trisomy 21 | 280.6 | 5.8 | 0.021 | | 8.9 | 0.032 | F |
| P3 | Trisomy 21 | 433.8 | 10.3 | 0.024 | | 36.7 | 0.085 | F |
| P4 | Trisomy 21 | 415.0 | 5.4 | 0.013 | | 21.1 | 0.051 | F |
| P5 | Trisomy 21 | 473.4 | 11.8 | 0.025 | | 34.7 | 0.073 | F |
| P6 | Trisomy 21 | 370.1 | 2.1 | 0.006 | | 13.5 | 0.037 | F |
| P7 | Trisomy 21 | 540.9 | 20.3 | 0.037 | | 51.0 | 0.094 | F |
| P8 | Trisomy 21 | 434.0 | 6.0 | 0.014 | | 19.2 | 0.044 | F |
| P9 | Trisomy 21 | 255.9 | 3.1 | 0.012 | | 10.2 | 0.040 | F |
| P10 | Trisomy 21 | 377.1 | 3.6 | 0.009 | | 28.9 | 0.077 | F |
| P11 | Trisomy 21 | 333.6 | 5.7 | 0.017 | | 41.1 | 0.123 | F |
| P12 | Trisomy 21 | 343.5 | 3.9 | 0.011 | | 11.0 | 0.032 | M |
| P13 | Trisomy 21 | 328.0 | 8.3 | 0.025 | | 28.1 | 0.086 | M |
| P14 | Trisomy 21 | 459.6 | 3.4 | 0.007 | | 29.5 | 0.064 | M |
| P15 | Trisomy 21 | 186.6 | 4.9 | 0.026 | | 15.1 | 0.081 | M |
| P16 | Trisomy 21 | 447.7 | 11.7 | 0.026 | | 38.0 | 0.085 | M |
| P17 | Trisomy 21 | 337.0 | 6.9 | 0.021 | | 24.6 | 0.073 | M |
| P18 | Trisomy 21 | 326.2 | 9.3 | 0.028 | | 30.6 | 0.094 | M |
| P19 | Normal | 199.2 | 1.0 | 0.005 | | 1.1 | 0.005 | F |
| P20 | Normal | 238.0 | 1.3 | 0.006 | | 3.0 | 0.012 | F |
| P21 | Normal | 209.6 | 1.3 | 0.006 | | 1.9 | 0.009 | F |
| P22 | Normal | 141.8 | 0.9 | 0.007 | | 1.6 | 0.011 | F |
| P23 | Normal | 103.6 | 1.5 | 0.014 | | 4.2 | 0.041 | F |
| P24 | Normal | 192.8 | 1.6 | 0.008 | | 3.0 | 0.016 | F |
| P25 | Normal | 138.0 | 1.6 | 0.011 | | 6.3 | 0.046 | F |
| P26 | Normal | 180.4 | 0.6 | 0.003 | | 4.6 | 0.025 | F |
| P27 | Normal | 100.4 | 0.6 | 0.006 | | 1.7 | 0.017 | F |
| P28 | Normal | 267.4 | 1.2 | 0.005 | | 3.5 | 0.013 | F |
| P29 | Normal | 186.2 | 1.2 | 0.007 | | 2.6 | 0.014 | F |
| P30 | Normal | 229.4 | 1.3 | 0.006 | | 4.1 | 0.018 | F |
| P31 | Normal | 361.6 | 1.8 | 0.005 | | 4.9 | 0.013 | F |
| P32 | Normal | 100.8 | 1.8 | 0.017 | | 2.1 | 0.021 | F |
| P33 | Normal | 116.2 | 1.3 | 0.011 | | 5.6 | 0.048 | F |
| P34 | Normal | 106.1 | 1.2 | 0.011 | | 5.8 | 0.055 | F |
| P35 | Normal | 164.3 | 0.4 | 0.002 | | 5.0 | 0.031 | F |
| P36 | Normal | 292.7 | 1.4 | 0.005 | | 6.3 | 0.021 | F |
| P37 | Normal | 215.0 | 0.5 | 0.002 | | 1.9 | 0.009 | F |
| P38 | Normal | 164.0 | 1.4 | 0.009 | | 3.6 | 0.022 | F |
| P39 | Normal | 251.6 | 2.0 | 0.008 | | 4.6 | 0.018 | F |
| P40 | Normal | 262.9 | 3.6 | 0.014 | | 11.4 | 0.044 | F |
| P41 | Normal | 345.9 | 3.0 | 0.009 | | 5.0 | 0.015 | F |
| P42 | Normal | 104.9 | 2.8 | 0.026 | | 13.4 | 0.128 | F |
| P43 | Normal | 163.0 | 1.3 | 0.008 | | 5.9 | 0.036 | F |
| P44 | Normal | 241.6 | 1.8 | 0.008 | | 12.1 | 0.050 | F |
| P45 | Normal | 148.5 | 2.3 | 0.015 | | 5.1 | 0.034 | F |
| P46 | Normal | 211.1 | 4.1 | 0.020 | | 8.6 | 0.041 | F |
| P47 | Normal | 167.2 | 2.4 | 0.014 | | 13.7 | 0.082 | F |
| P48 | Normal | 132.2 | 1.1 | 0.008 | | 10.2 | 0.077 | F |
| P49 | Normal | 175.9 | 2.8 | 0.016 | | 10.0 | 0.057 | F |
| P50 | Normal | 149.5 | 1.6 | 0.011 | | 4.0 | 0.027 | F |
| P51 | Normal | 262.7 | 1.7 | 0.006 | | 5.7 | 0.022 | F |
| P52 | Normal | 127.9 | 3.4 | 0.026 | | 8.2 | 0.064 | F |
| P53 | Normal | 254.6 | 2.1 | 0.008 | | 17.8 | 0.070 | F |
| P54 | Normal | 183.2 | 3.2 | 0.017 | | 10.0 | 0.055 | F |
| P55 | Normal | 351.8 | 1.6 | 0.005 | | 10.2 | 0.029 | F |
| P56 | Normal | 183.7 | 3.5 | 0.019 | | 16.0 | 0.087 | F |
| P57 | Normal | 183.1 | 3.6 | 0.020 | | 10.1 | 0.055 | F |
| P58 | Normal | 136.8 | 2.7 | 0.020 | | 12.3 | 0.090 | F |
| P59 | Normal | 286.4 | 1.4 | 0.005 | | 7.9 | 0.028 | F |
| P60 | Normal | 202.3 | 3.6 | 0.018 | | 16.3 | 0.081 | F |
| P61 | Normal | 181.9 | 3.4 | 0.019 | | 14.5 | 0.080 | F |
| P62 | Normal | 105.6 | 0.6 | 0.005 | | 1.7 | 0.016 | F |
| P63 | Normal | 301.3 | 1.6 | 0.005 | | 4.5 | 0.015 | F |
| P64 | Normal | 236.6 | 1.7 | 0.007 | | 10.3 | 0.044 | F |
| P65 | Normal | 240.8 | 1.1 | 0.005 | | 4.9 | 0.020 | M |
| P66 | Normal | 167.0 | 1.7 | 0.010 | | 3.5 | 0.021 | M |
| P67 | Normal | 199.2 | 0.9 | 0.005 | | 1.1 | 0.006 | M |
| P68 | Normal | 192.0 | 0.7 | 0.004 | | 2.1 | 0.011 | M |
| P69 | Normal | 120.4 | 1.8 | 0.015 | | 5.1 | 0.042 | M |
| P70 | Normal | 120.4 | 1.9 | 0.016 | | 5.1 | 0.043 | M |
| P71 | Normal | 139.0 | 0.9 | 0.006 | | 3.2 | 0.023 | M |
| P72 | Normal | 147.2 | 1.9 | 0.013 | | 3.9 | 0.026 | M |
| P73 | Normal | 124.4 | 1.7 | 0.014 | | 3.9 | 0.031 | M |
| P74 | Normal | 107.8 | 0.9 | 0.008 | | 1.9 | 0.017 | M |
| P75 | Normal | 127.8 | 0.5 | 0.004 | | 1.8 | 0.014 | M |
| P76 | Normal | 317.1 | 5.6 | 0.018 | | 3.9 | 0.012 | M |
| P77 | Normal | 136.9 | 3.2 | 0.024 | | 11.3 | 0.083 | M |
| P78 | Normal | 113.6 | 3.4 | 0.029 | | 10.4 | 0.092 | M |
| P79 | Normal | 296.1 | 1.7 | 0.006 | | 9.8 | 0.033 | M |
| P80 | Normal | 217.8 | 3.3 | 0.015 | | 5.3 | 0.024 | M |
| P81 | Normal | 314.4 | 4.4 | 0.014 | | 8.4 | 0.027 | M |
| P82 | Normal | 199.4 | 3.0 | 0.015 | | 4.4 | 0.022 | M |
| P83 | Normal | 242.6 | 3.6 | 0.015 | | 6.9 | 0.028 | M |
| P84 | Normal | 314.2 | 3.4 | 0.011 | | 13.1 | 0.042 | M |
| P85 | Normal | 211.8 | 4.9 | 0.023 | | 9.4 | 0.044 | M |
| P86 | Normal | 381.1 | 0.7 | 0.002 | | 6.7 | 0.018 | M |
| P87 | Normal | 224.5 | 1.7 | 0.008 | | 8.7 | 0.039 | M |
| P88 | Normal | 178.2 | 4.9 | 0.027 | | 17.2 | 0.096 | M |
| P89 | Normal | 288.1 | 4.2 | 0.015 | | 9.8 | 0.034 | M |
| P90 | Normal | 295.7 | 3.6 | 0.012 | | 7.5 | 0.025 | M |
| P91 | Normal | 290.9 | 1.7 | 0.006 | | 13.0 | 0.045 | M |
| P92 | Normal | 155.1 | 4.0 | 0.026 | | 10.5 | 0.067 | M |
| P93 | Normal | 112.1 | 2.4 | 0.022 | | 10.5 | 0.093 | M |
| P94 | Normal | 291.4 | 3.9 | 0.013 | | 14.5 | 0.050 | M |
| P95 | Normal | 187.8 | 5.1 | 0.027 | | 13.0 | 0.069 | M |
| P96 | Normal | 137.6 | 3.7 | 0.027 | | 5.5 | 0.040 | M |
| P97 | Normal | 143.4 | 2.9 | 0.021 | | 11.9 | 0.083 | M |
| P98 | Normal | 366.5 | 2.1 | 0.006 | | 5.8 | 0.016 | M |
| P99 | Normal | 213.6 | 3.0 | 0.014 | | 2.9 | 0.014 | M |
| P100 | Normal | 196.5 | 4.7 | 0.024 | | 14.9 | 0.076 | M |
| P101 | Normal | 316.4 | 1.4 | 0.004 | | 5.9 | 0.019 | M |
| P102 | Normal | 159.4 | 1.5 | 0.010 | | 3.5 | 0.022 | M |
| P103 | Normal | 289.9 | 6.9 | 0.024 | | 15.5 | 0.053 | M |
| P104 | Normal | 178.4 | 3.9 | 0.022 | | 9.7 | 0.055 | M |
| P105 | Normal | 286.4 | 5.7 | 0.020 | | 10.0 | 0.035 | M |
| P106 | Normal | 265.8 | 4.7 | 0.018 | | 10.2 | 0.039 | M |
| P107 | Normal | 308.4 | 9.7 | 0.032 | | 10.5 | 0.034 | M |
| P108 | Normal | 213.5 | 4.6 | 0.021 | | 11.5 | 0.054 | M |
