# Supplementary material for: Non-Invasive Epigenetic Detection of Fetal Trisomy 21 in First Trimester Maternal Plasma
Source: PLoS One. 2011 Nov 23;6(11):e27709. doi: 10.1371/journal.pone.0027709 (PMC3223183; doi:10.1371/journal.pone.0027709)
Supplement: Table S3 — Methylated PDE9A levels obtained from 108 samples. (DOC) [file pone.0027709.s004.doc]

**Supplementary Table 3.** Methylated *PDE9A* levels obtained from the 108 samples

| Sample | Status | Level | inter SD | inter CV | intra SD | intra CV | Gender |
| --- | --- | --- | --- | --- | --- | --- | --- |
| P1 | Trisomy 21 | 2170.2 | 75.8 | 0.035 | 54.4 | 0.025 | F |
| P2 | Trisomy 21 | 1427.4 | 50.7 | 0.036 | 62.4 | 0.044 | F |
| P3 | Trisomy 21 | 2526.4 | 37.3 | 0.015 | 70.1 | 0.028 | F |
| P4 | Trisomy 21 | 1733.7 | 61.4 | 0.035 | 68.8 | 0.040 | F |
| P5 | Trisomy 21 | 2602.9 | 68.4 | 0.026 | 99.3 | 0.038 | F |
| P6 | Trisomy 21 | 1752.5 | 65.7 | 0.038 | 77.9 | 0.044 | F |
| P7 | Trisomy 21 | 2446.8 | 64.8 | 0.026 | 49.4 | 0.020 | F |
| P8 | Trisomy 21 | 2135.8 | 86.8 | 0.041 | 51.4 | 0.024 | F |
| P9 | Trisomy 21 | 1722.0 | 53.5 | 0.031 | 51.7 | 0.030 | F |
| P10 | Trisomy 21 | 1609.8 | 55.5 | 0.035 | 76.1 | 0.047 | F |
| P11 | Trisomy 21 | 1666.2 | 33.2 | 0.020 | 38.4 | 0.023 | F |
| P12 | Trisomy 21 | 1614.7 | 69.7 | 0.043 | 57.1 | 0.035 | M |
| P13 | Trisomy 21 | 1583.8 | 52.7 | 0.033 | 52.0 | 0.033 | M |
| P14 | Trisomy 21 | 2446.1 | 79.0 | 0.032 | 76.6 | 0.031 | M |
| P15 | Trisomy 21 | 1956.0 | 72.1 | 0.037 | 58.3 | 0.030 | M |
| P16 | Trisomy 21 | 2489.4 | 44.5 | 0.018 | 51.6 | 0.021 | M |
| P17 | Trisomy 21 | 1998.8 | 52.3 | 0.026 | 64.6 | 0.032 | M |
| P18 | Trisomy 21 | 1715.5 | 43.8 | 0.026 | 62.4 | 0.036 | M |
| P19 | Normal | 2907.5 | 67.2 | 0.023 | 64.0 | 0.022 | F |
| P20 | Normal | 2352.0 | 62.1 | 0.026 | 15.9 | 0.007 | F |
| P21 | Normal | 1828.3 | 54.4 | 0.030 | 36.1 | 0.020 | F |
| P22 | Normal | 2436.2 | 89.7 | 0.037 | 71.1 | 0.029 | F |
| P23 | Normal | 1694.5 | 72.0 | 0.042 | 71.2 | 0.042 | F |
| P24 | Normal | 1548.8 | 81.6 | 0.053 | 76.7 | 0.050 | F |
| P25 | Normal | 1958.6 | 71.0 | 0.036 | 50.0 | 0.026 | F |
| P26 | Normal | 2444.2 | 30.7 | 0.013 | 33.4 | 0.014 | F |
| P27 | Normal | 1247.1 | 50.8 | 0.041 | 34.9 | 0.028 | F |
| P28 | Normal | 2174.4 | 70.6 | 0.032 | 75.5 | 0.035 | F |
| P29 | Normal | 2138.2 | 71.6 | 0.033 | 69.7 | 0.033 | F |
| P30 | Normal | 2624.0 | 73.0 | 0.028 | 103.2 | 0.039 | F |
| P31 | Normal | 2272.8 | 77.9 | 0.034 | 67.4 | 0.030 | F |
| P32 | Normal | 1965.7 | 47.7 | 0.024 | 54.4 | 0.028 | F |
| P33 | Normal | 2264.9 | 64.7 | 0.029 | 76.5 | 0.034 | F |
| P34 | Normal | 2023.1 | 68.9 | 0.034 | 79.9 | 0.040 | F |
| P35 | Normal | 2907.5 | 62.2 | 0.021 | 70.6 | 0.024 | F |
| P36 | Normal | 1564.5 | 42.2 | 0.027 | 42.5 | 0.027 | F |
| P37 | Normal | 2060.6 | 25.3 | 0.012 | 20.7 | 0.010 | F |
| P38 | Normal | 2309.2 | 14.6 | 0.006 | 42.7 | 0.018 | F |
| P39 | Normal | 2106.3 | 50.8 | 0.024 | 51.1 | 0.024 | F |
| P40 | Normal | 2209.0 | 78.8 | 0.036 | 69.8 | 0.032 | F |
| P41 | Normal | 2432.9 | 38.8 | 0.016 | 116.5 | 0.048 | F |
| P42 | Normal | 1277.9 | 29.5 | 0.023 | 30.2 | 0.024 | F |
| P43 | Normal | 1979.2 | 16.5 | 0.008 | 54.4 | 0.028 | F |
| P44 | Normal | 1737.7 | 41.4 | 0.024 | 36.9 | 0.021 | F |
| P45 | Normal | 1706.6 | 35.4 | 0.021 | 77.4 | 0.045 | F |
| P46 | Normal | 2366.9 | 39.5 | 0.017 | 75.1 | 0.032 | F |
| P47 | Normal | 1835.7 | 37.8 | 0.021 | 42.7 | 0.023 | F |
| P48 | Normal | 1746.2 | 25.2 | 0.014 | 34.8 | 0.020 | F |
| P49 | Normal | 1878.9 | 59.0 | 0.031 | 26.3 | 0.014 | F |
| P50 | Normal | 1503.2 | 17.8 | 0.012 | 24.9 | 0.017 | F |
| P51 | Normal | 1564.3 | 50.0 | 0.032 | 58.4 | 0.037 | F |
| P52 | Normal | 1630.1 | 51.8 | 0.032 | 29.5 | 0.018 | F |
| P53 | Normal | 3044.8 | 77.4 | 0.025 | 46.7 | 0.015 | F |
| P54 | Normal | 1571.2 | 29.5 | 0.019 | 62.5 | 0.040 | F |
| P55 | Normal | 2417.1 | 66.6 | 0.028 | 75.5 | 0.031 | F |
| P56 | Normal | 2311.3 | 75.1 | 0.032 | 95.0 | 0.041 | F |
| P57 | Normal | 1607.4 | 59.1 | 0.037 | 61.8 | 0.038 | F |
| P58 | Normal | 1288.2 | 20.9 | 0.016 | 15.3 | 0.012 | F |
| P59 | Normal | 2056.2 | 36.7 | 0.018 | 48.2 | 0.023 | F |
| P60 | Normal | 1431.3 | 34.9 | 0.024 | 43.5 | 0.030 | F |
| P61 | Normal | 2151.7 | 78.9 | 0.037 | 12.8 | 0.006 | F |
| P62 | Normal | 1633.8 | 20.0 | 0.012 | 48.8 | 0.030 | F |
| P63 | Normal | 2129.6 | 70.5 | 0.033 | 55.7 | 0.026 | F |
| P64 | Normal | 2277.9 | 37.1 | 0.016 | 86.5 | 0.038 | F |
| P65 | Normal | 2419.5 | 35.6 | 0.015 | 85.0 | 0.035 | M |
| P66 | Normal | 2959.0 | 80.4 | 0.027 | 44.5 | 0.015 | M |
| P67 | Normal | 1687.5 | 23.0 | 0.014 | 77.0 | 0.046 | M |
| P68 | Normal | 1529.1 | 44.1 | 0.029 | 59.7 | 0.039 | M |
| P69 | Normal | 1944.4 | 76.8 | 0.040 | 85.4 | 0.044 | M |
| P70 | Normal | 1856.3 | 48.7 | 0.026 | 34.6 | 0.019 | M |
| P71 | Normal | 2014.0 | 26.5 | 0.013 | 99.0 | 0.049 | M |
| P72 | Normal | 2120.0 | 33.8 | 0.016 | 66.4 | 0.031 | M |
| P73 | Normal | 1571.2 | 156.9 | 0.100 | 68.7 | 0.044 | M |
| P74 | Normal | 1598.5 | 50.4 | 0.032 | 13.0 | 0.008 | M |
| P75 | Normal | 1197.9 | 11.5 | 0.010 | 26.0 | 0.022 | M |
| P76 | Normal | 1814.1 | 80.1 | 0.044 | 64.1 | 0.035 | M |
| P77 | Normal | 2645.3 | 130.6 | 0.049 | 91.0 | 0.034 | M |
| P78 | Normal | 1997.8 | 42.5 | 0.021 | 55.8 | 0.028 | M |
| P79 | Normal | 2982.7 | 65.5 | 0.022 | 74.6 | 0.025 | M |
| P80 | Normal | 1776.3 | 33.7 | 0.019 | 52.8 | 0.030 | M |
| P81 | Normal | 2333.8 | 29.0 | 0.012 | 65.9 | 0.028 | M |
| P82 | Normal | 1527.1 | 24.1 | 0.016 | 32.8 | 0.021 | M |
| P83 | Normal | 1638.1 | 46.8 | 0.029 | 58.1 | 0.035 | M |
| P84 | Normal | 1592.5 | 48.8 | 0.031 | 63.7 | 0.040 | M |
| P85 | Normal | 2544.0 | 69.1 | 0.027 | 34.4 | 0.014 | M |
| P86 | Normal | 2411.2 | 53.9 | 0.022 | 36.7 | 0.015 | M |
| P87 | Normal | 2710.6 | 84.7 | 0.031 | 54.7 | 0.020 | M |
| P88 | Normal | 2011.1 | 98.1 | 0.049 | 66.1 | 0.033 | M |
| P89 | Normal | 2114.7 | 32.7 | 0.015 | 62.2 | 0.029 | M |
| P90 | Normal | 2138.2 | 21.8 | 0.010 | 14.0 | 0.007 | M |
| P91 | Normal | 2917.1 | 56.4 | 0.019 | 82.0 | 0.028 | M |
| P92 | Normal | 1414.6 | 17.5 | 0.012 | 33.2 | 0.023 | M |
| P93 | Normal | 1506.6 | 50.7 | 0.034 | 47.0 | 0.031 | M |
| P94 | Normal | 2444.2 | 101.6 | 0.042 | 61.8 | 0.025 | M |
| P95 | Normal | 1573.6 | 48.5 | 0.031 | 70.6 | 0.045 | M |
| P96 | Normal | 1250.1 | 11.5 | 0.009 | 13.8 | 0.011 | M |
| P97 | Normal | 1556.3 | 39.7 | 0.026 | 21.9 | 0.014 | M |
| P98 | Normal | 2023.7 | 44.7 | 0.022 | 27.2 | 0.013 | M |
| P99 | Normal | 1609.6 | 35.0 | 0.022 | 38.8 | 0.024 | M |
| P100 | Normal | 1312.8 | 52.8 | 0.040 | 19.7 | 0.015 | M |
| P101 | Normal | 2513.1 | 54.8 | 0.022 | 40.4 | 0.016 | M |
| P102 | Normal | 1450.8 | 25.1 | 0.017 | 42.7 | 0.029 | M |
| P103 | Normal | 1877.3 | 59.9 | 0.032 | 60.7 | 0.032 | M |
| P104 | Normal | 1748.6 | 5.7 | 0.003 | 22.1 | 0.013 | M |
| P105 | Normal | 2089.7 | 54.1 | 0.026 | 68.2 | 0.033 | M |
| P106 | Normal | 2064.9 | 38.7 | 0.019 | 70.0 | 0.034 | M |
| P107 | Normal | 2855.5 | 27.8 | 0.010 | 38.0 | 0.013 | M |
| P108 | Normal | 2297.9 | 124.9 | 0.054 | 39.2 | 0.017 | M |
